# Molecular Dynamics Simulation on Thickening and Solubility Properties of Novel Thickener in Supercritical Carbon Dioxide

**DOI:** 10.3390/molecules29112529

**Published:** 2024-05-27

**Authors:** Xiaohui Wang, Shiwei Liang, Qihong Zhang, Tianjiao Wang, Xiao Zhang

**Affiliations:** 1 Beijing Key Laboratory of Optical Detection Technology for Oil and Gas, China University of Petroleum-Beijing, Beijing 102249, China; wangxiaohui@cup.edu.cn (X.W.); 2023211325@student.cup.edu.cn (S.L.); 2021211324@student.cup.edu.cn (Q.Z.); 2023216161@student.cup.edu.cn (T.W.); 2 National Key Laboratory of Petroleum Resources and Engineering, China University of Petroleum-Beijing, Beijing 102249, China; 3 College of Science, China University of Petroleum, Beijing 102249, China

**Keywords:** thickener, supercritical carbon dioxide, viscosity, molecular dynamics

## Abstract

Supercritical CO_2_ has wide application in enhancing oil recovery, but the low viscosity of liquid CO_2_ can lead to issues such as poor proppant-carrying ability and high filtration loss. Therefore, the addition of thickening agents to CO_2_ is vital. Hydrocarbon polymers, as a class of green and sustainable materials, hold tremendous potential for acting as thickeners in supercritical CO_2_ systems, and PVAc is one of the best-performing hydrocarbon thickeners. To further improve the viscosity enhancement and solubility of PVAc, here we designed a novel polymer structure, PVAO, by introducing CO_2_-affine functional groups to PVAc. Molecular dynamics simulations were adopted to analyze viscosity and relevant solubility parameters systematically. We found that PVAO exhibits superior performance, with a viscosity enhancement of 1.5 times that of PVAc in supercritical CO_2_. While in the meantime, PVAO maintains better solubility characteristics than PVAc. Our findings offer insights for the future design of other high-performance polymers.

## 1. Introduction

With the huge growth of energy demand in the world, enhanced oil recovery (EOR) has received considerable attention in the petroleum industry. Currently, most easily accessible oil reservoirs have been drilled, leading to an overall decline in oil production. Therefore, maximizing the utilization of known resources becomes more practical than exploring new oil wells; thus, EOR has become increasingly crucial.

In crude oil recovery, three processes are commonly employed. Primary oil recovery involves the extraction of oil under its own pressure and through gas expansion by dissolution, accounting for 5–20% of oil recovery. Secondary oil recovery employs water flooding to displace oil. In recent decades, hydraulic fracturing technology has emerged as an effective method for oil and gas production enhancement and has been widely utilized. However, hydraulic fracturing technology has its drawbacks, which include significant water resource consumption, potential damage to reservoirs, and the risk of groundwater contamination due to the addition of various chemical substances into the injected water. Additionally, even after primary and secondary oil recovery, over half of the oil remains trapped in the reservoir. To further increase production, the implementation of more advanced EOR techniques, commonly referred to as tertiary oil recovery, is necessary. EOR is a method of injecting displacing agents into reservoirs to improve the physical and chemical characteristics of the reservoir and its fluids, thereby enhancing oil displacement efficiency. With the implementation of tertiary oil recovery, the field’s utilization rate can reach around 70%.

Among the various displacing agents, supercritical CO_2_ (scCO_2_) has garnered significant attention due to its excellent performance as an oil-displacing agent [1,2]. ScCO_2_ exhibits intermediate properties between those of gas and liquid. It has obvious characteristics such as high diffusivity, low viscosity, low surface tension, and controllable solubility. In oil recovery, compared to traditional water flooding, scCO_2_ has significant advantages. Firstly, scCO_2_ possesses strong fracturing capability and is easily displaced [2], and it is also applicable to various types of reservoirs [3]. Secondly, with low raw material costs, the critical temperature and pressure of scCO_2_ are 304.1 K and 7.38 MPa, respectively, which are lower than the temperature and pressure within the reservoir [4,5]. Therefore, carbon dioxide can be transformed into a supercritical liquid once entering the reservoir, saving the energy required for its conversion. Thirdly, scCO_2_ can be converted into gas form and expelled from the reservoir after fracturing, causing no damage to the rock formation and preventing expansion; thus, it is non-toxic, non-polluting, non-flammable, and recyclable [6]. As an emerging oil and gas production method, scCO_2_ fracturing technology exhibits significant advantages in terms of environmental friendliness, efficiency, and adaptability. It is expected to play an increasingly important role in future oil and gas development [7,8,9].

However, the low-viscosity nature of scCO_2_ gives rise to challenges such as viscosity fingering, limited sand-carrying capacity, filter loss, and reduced efficiency in oil and gas recovery. To overcome these challenges, thickeners are required to enhance the viscosity of scCO_2_. In recent years, researchers have been devoted to the design of novel CO_2_-responsive polymers, and the investigation of CO_2_ thickening agents has undergone several stages. Girard and Mertsch separately discovered that fluorine-containing polymers and silicon-based materials exhibit excellent solubility in CO_2_, leading to a significant increase in viscosity [10,11,12]. However, the high costs of fluorine-containing and silicon-based polymers pose challenges for their large-scale industrial utilization as CO_2_-responsive materials. Additionally, when fluorine-containing polymers are used in the field of oilfield chemistry, fluorine polymers are often discharged into the environment with wastewater, causing irreparable environmentally damage as they are non-biodegradable. As a result, the design of fluorine-free polymers composed solely of carbon (C), hydrogen (H), and oxygen (O) atoms, known as hydrocarbon polymers, has gained more attention. Extensive experimental studies have demonstrated the viability of several hydrocarbon polymers, such as poly (vinyl acetate) (PVAc), poly (vinyl ethyl ether) (PVEE), poly (propylene oxide) (PPO), and poly (vinyl methoxymethyl ether). Among these, PVAc is the most promising polymer due to its relatively high solubility in CO_2_, which constitutes its main advantage over other materials [13].

Yet hydrocarbon polymers represented by PVAc still do not have the expected thickening performance compared with other thickeners, such as fluorine-containing polymers. To further improve the viscosity enhancement and solubility of PVAc, in this study, we designed a novel polymer structure, PVAO, by introducing CO_2_-affine functional groups to PVAc. Molecular dynamics (MD) simulations were adopted to analyze viscosity and relevant solubility parameters systematically. Viscosity, radial distribution function, interaction energy, cohesive energy density, and solubility parameters are given. We found that PVAO exhibits superior performance both in viscosity enhancement and solubility characteristics than PVAc.

## 2. Results and Discussion

### 2.1. The Thickening Effect of PVAO

Predicting the viscosity of supercritical CO_2_ remains a crucial task. Classical MD simulations have been employed for shear viscosity predictions. The primary methods utilized include non-equilibrium molecular dynamics (NEMD) and equilibrium molecular dynamics (EMD), with the Green–Kubo method based on EMD, which is the most widely applied approach [14]. Here, we present equilibrium MD calculations for the viscosity of pure scCO_2_, PVAc/CO_2_, and PVAO/CO_2_ using the standard Green–Kubo method.

In the Green–Kubo theory, shear viscosity is calculated from the integral over time of the pressure tensor autocorrelation function [14], as follows:

(1) $$\eta =\frac{V}{K_{B}T}\int_{0}^{\infty }\langle P_{\alpha \beta }\left(t\right)\cdot P_{\alpha \beta }\left(0\right)\rangle dt$$

where $K_{B}$ is the Boltzmann constant, T is the absolute temperature, t is time, V is the volume of the simulation box, and $P_{\alpha \beta }$ denotes the element αβ of the pressure tensor. The symmetry of the cubic simulation box implies that the three directions, x, y, and z, are equivalent. Theoretically, the autocorrelation function of the stress tensor should decay to zero as time progresses. Then, we can obtain a constant value that corresponds to the computed shear viscosity using Equation (1).

Yong Zhang et al. proposed a method for calculating shear viscosity by executing multiple independent trajectories and taking the average of the running time integrals. In order to calculate the viscosity, five independent trajectories were generated using different initial velocity distribution seeds, each with a length of 300 ps. Based on these trajectories, the average shear viscosity of each system at different temperatures was calculated using Equation (1) [15].

To validate the rationality of the methods, the shear viscosity of supercritical CO_2_ was calculated using the proposed method under the conditions of 23 °C and 20 MPa. The simulation results demonstrated a viscosity value of 0.087 cp, which closely matched the experimental measurement of 0.094 cp [16]. This result serves as evidence supporting the feasibility of the employed methodology.

**Figure 1:**
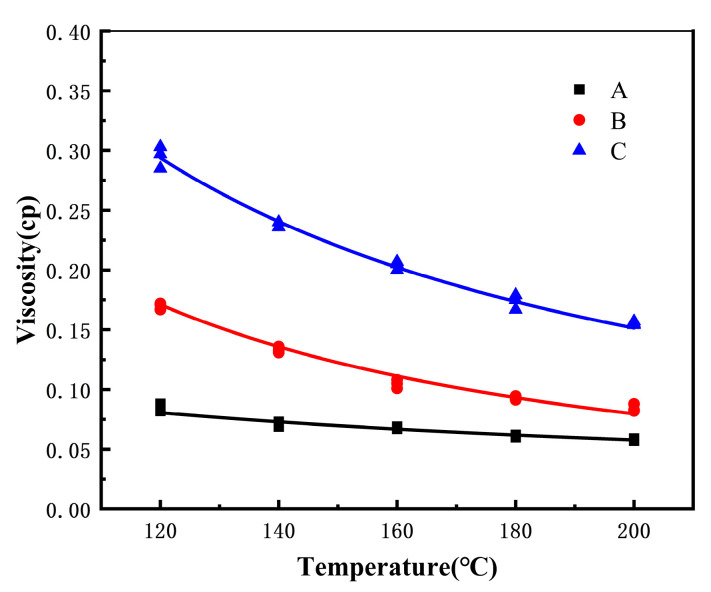
The viscosities of (**A**) scCO_2_; (**B**) scCO_2_ with one PVAc chain; and (**C**) scCO_2_ with one PVAO chain with a pressure of 60 MPa and a temperature between 120 and 200 °C.

For the thickening effect of PVAO, the crucial aspect lies in determining whether the novel thickening agent exhibits superior viscosity enhancement compared to PVAc. Figure 1 presents a comparative analysis of the viscosity enhancement effects between PVAO and PVAc under identical conditions.

Figure 1 shows the viscosity of (A) scCO_2_ with a pressure of 60 MPa and a temperature between 120 and 200 °C, (B) scCO_2_ with one PVAc chain, and (C) scCO_2_ with one PVAO chain. As shown in Figure 1, as the temperature increased, the viscosities of the three systems gradually decreased. Moreover, the viscosity-enhancing effect of PVAO was much stronger than that of PVAc. Under 120 °C and 60 MPa, the addition of a PVAO chain in scCO_2_ fluid led to a significant increase in viscosity to 0.245 cp, approximately three times higher than that of pure CO_2_ fluid, while under the same temperature and pressure conditions, the viscosity of scCO_2_ with a PVAc chain was approximately twice that of pure scCO_2_. PVAO is a promising hydrocarbon polymer with higher viscosity-enhancing efficiency. The reason for the significantly higher viscosity enhancement of PVAO compared to PVAc may be the fact that, at similar weight fractions, PVAO contains a greater number of key functional groups, which enhances the interactions between the C atoms in CO_2_ and the O atoms in the branched chain of PVAO, thus contributing to its thickening effect.

### 2.2. Diffusivity

Mean square displacement (MSD) refers to the deviation of particle positions from a reference point with time. As the observation time approaches infinity, MSD becomes directly proportional to the observation time limit. In scCO_2_ fluid systems, the MSD of CO_2_ within a certain range of polymers exhibits a linear relationship with time evolution. Moreover, the slope of this relationship is related to the diffusion coefficient D, as expressed by the following formula [17]:

(2) $$\mathrm{MSD}=\langle {\left|X_{i}\left(t_{0}+t\right)-X_{i}\left(t_{0}\right)\right|}^{2}\rangle$$

(3) $$D=\frac{1}{6N}\underset{t\to \infty }{\lim}\frac{d}{dt}\sum \langle {\left|X_{i}\left(t_{0}+t\right)-X_{i}\left(t_{0}\right)\right|}^{2}\rangle$$

**Figure 2:**
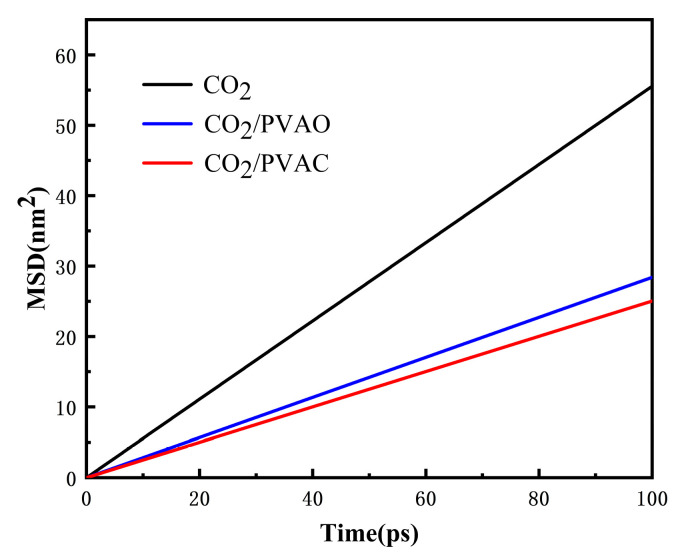
MSD–time curves of scCO_2_ (black line), scCO_2_ with PVAO (blue line), and scCO_2_ with PVAc (red line).

The diffusion coefficient characterizes the extent of molecular diffusion in liquids, indicating the speed of molecular diffusion. The MSD curve can be obtained through MD simulations, which is shown in Figure 2. The slope of the curve can be determined by linear fitting. By comparing the magnitude of the diffusion coefficients, the strength of the interaction between polymers and CO_2_ can be roughly estimated. The obtained curve clearly showed that the diffusion coefficients of the scCO_2_ systems with PVAc and PVAO were smaller than those in an scCO_2_ system without thickeners. The diffusion coefficients for the CO_2_, PVAc/CO_2_, and PVAO/CO_2_ systems were denoted as 9.26 ± 0.069 × 10^−7^ cm^2^/s, 3.88 ± 0.073 × 10^−7^ cm^2^/s, and 4.58 ± 0.058 × 10^−7^ cm^2^/s, respectively. This result indicated the ability of PVAO to bind CO_2_ molecules, which reflected the thickening effects of PVAO and PVAc.

### 2.3. Radial Distribution Function

The presence of lone pair electrons on O in CO_2_ and the Lewis acid–Lewis base (LA–LB) interaction between the O atoms in PVAO and the C atoms in CO_2_ are the primary influencing factors for the dissolution of ether-based and carbonyl-containing polymers in carbon dioxide [18,19], and the interaction between molecules or atoms can be described by the radial distribution function (RDF) [20,21].

RDF can be obtained by performing MD simulations using the *Forcite* module in Materials Studio, and it represents the relative local density of atom B with respect to the bulk density in a region around a central atom A, within a distance radius of r [22]. In essence, the RDF is a probability calculation that determines the likelihood of finding another atom at a distance of r from the reference atom. RDF can be denoted by g(r, r’). For small values of |r − r’|, g(r, r’) primarily characterizes the atomic packing and distances between bonds. For long-range situations, since the probability of finding an atom is approximately the same for a given distance, g(r, r’) becomes flat and ultimately approaches a constant value as |r − r’| increases. Typically, when defining g(r, r’), it is normalized such that g(r, r’) approaches 1 as |r − r’| tends to infinity. The formula for g(r, r’) is as follows [23]:

(4) $$g\left(r\right)=\frac{\mathrm{dN}}{4\rho \pi^{2}}$$

**Figure 3:**
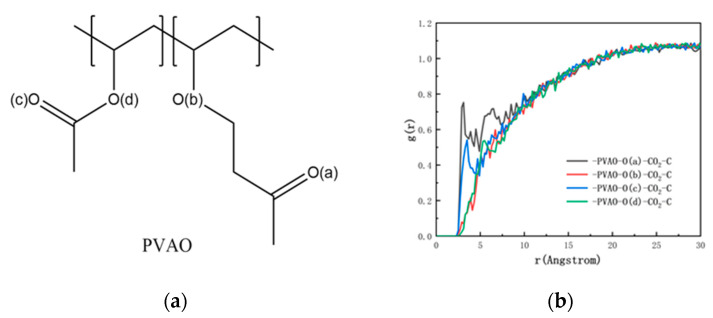
(**a**) Numbering diagram of O atoms in PVAO. (**b**) RDF between different O atoms in PVAO and C atoms in CO_2_.

The integrated RDF between the oxygen atom in PVAO and the carbon atom in CO_2_ was calculated and is illustrated in Figure 3b, and the specified oxygen atom in PVAO is labeled in Figure 3a. As shown in Figure 3b, both the O(a) and O(c) atoms in PVAO exhibited pronounced peaks in their RDF curves with respect to the C atoms in CO_2_, whereas O(b) and O(d) displayed no significant peak features in the RDF curves. The results indicated the presence of LA–LB interactions between O(a) and O(c) in PVAO and the C atoms in CO_2_, and that the LA–LB interactions between the carbonyl oxygen atom at the distal end of the PVAO side chain and the carbon atom in CO_2_ were much stronger than the LA–LB interactions involving the oxygen atom at the proximal end. The insights derived from the RDF simulation results offer valuable guidance for the design of novel polymer structures. It was observed that the addition of carbonyl oxygen atoms at the distal end, as opposed to those in close proximity, was more likely to enhance the solubility of the polymer in CO_2_.

### 2.4. Interaction Energy

Interaction energy is the difference between the energy of the complex minus the energy of the isolated monomers in the complex. The lower the interaction energy, the more stable the structure. For the CO_2_–polymer chain system, the interaction energy can be written as [20,21]:

(5) $$E_{inter}=E_{CO_{2}-chain}-E_{CO_{2}}-E_{chain}$$

In the above equation, $E_{inter}$ represents the interaction energy between CO_2_ and the polymer chain,${\;E}_{CO_{2}-chain}\;$denotes the total energy of the CO_2_–polymer system, $E_{CO_{2}}\;$and$\;E_{chain}$ are the energies of CO_2_ and the polymer chain, respectively.

In order to assess the polymer–CO_2_ interactions, MD simulations were performed in the NPT ensemble. As shown in Table 1, the interaction energy of the PVAO-thickened CO_2_ system was calculated to be −493.1 KJ/mol, while that of the PVAc-thickened CO_2_ system was −474.6 KJ/mol, which was slightly higher than the former, indicating a lower stability compared to the PVAO-thickened CO_2_ system. The standard deviations of the interaction energies for the two systems were separately computed over the last 10 frames. The standard deviations of PVAc/CO_2_ and PVAO/CO_2_ were 7.79 and 8.81 kJ/mol, respectively. The difference in the interaction energies can serve as a basis for evaluating the difference in solubility within the error bars. Thus, PVAO may have better compatibility with CO_2_ and could be a more suitable polymer for CO_2_ affinity. Moreover, these results indicated that the solubility of the PVAO chain in practical applications may not be weaker than that of PVAc.

However, it should be noted that, in addition to the interaction energy between CO_2_ and the polymer chain, intermolecular interactions between polymer chains are also an important factor affecting solubility [20]. In order to achieve a more comprehensive prediction of solubility, it is necessary to incorporate cohesive energy density and solubility parameters.

### 2.5. Cohesive Energy Density and Solubility Parameters

Cohesive energy density (CED) and solubility parameters are quantitative measures used to characterize intermolecular interactions between molecules. CED is employed specifically for evaluating non-covalent bonding interactions quantitatively, which can be calculated by considering parameters such as partial charge distributions and atomic distances within a molecule. Accurate computation of CED ($\;e_{coh}\;$) holds significant importance in predicting molecular properties and reactivity, which can be utilized as descriptors to characterize both compatibility and solubility properties within the system under investigation [24]. Meanwhile, solubility parameters primarily describe solubility and compatibility. The expression for solubility parameter δ is given by the following equation: $\delta =\sqrt{e_{coh}}$. $e_{coh}\;$, and δ can serve as a basis for evaluating the molecular forces between polymer chains and also for evaluating the solubility of thickeners in scCO_2_ [3,21]. The analysis of cohesive energy density required the utilization of the *Forcite* module within the MS software. The cohesive energy density was computed for the last 10 frames of the trajectory, and the average value was obtained. In order to investigate the internal interactions within polymer chains, the cohesive energy density and solubility parameters under 120 ℃ and 60 MPa conditions were calculated and are listed in Table 2. Each system consisted of three polymer chains, with the composition of each polymer chain outlined in Table 2.

From Table 2, we can see that PVAc possessed the highest cohesive energy density and solubility parameters, indicating the strongest interactions between PVAc chains compared with the pure CO_2_ and PVAO systems. The intensified interactions could result in increased interfacial tension, which might hinder the blending process between PVAc and CO_2_. In addition, PVAO exhibited relatively lower cohesive energy density and milder interactions between internal polymer chains. The blending process between polymers is essentially a diffusion process between molecular chains, constrained by the interactions between the chains. The compatibility between different components can also be assessed by the difference in solubility parameter δ. When the δ values are closer, better compatibility can be observed, which follows the theory of similar dissolves mutually. The difference in solubility parameter (|Δδ|) between the PVAO polymer and CO_2_ was smaller than that between PVAc and CO_2_, indicating that PVAO has merits over PVAc in terms of solubility.

## 3. Simulation Details and Methods

Qin et al. explored the structural and dynamic characteristics of scCO_2_ fluids on hydroxylated and methylated amorphous silica surfaces using MD simulations [25]. Hu et al. investigated the interaction mechanisms between various functional groups and scCO_2_ through MD simulations [20,21]. These studies undoubtedly demonstrate that MD simulations serve as powerful tools for investigating scCO_2_ at the molecular level.

In our study, MD simulations were performed using Material Studio 8.0 developed by Accelrys [26]. The commonly used force fields for simulating polymers are AMBER [27], CHARMM [28], COMPASS [29,30], etc. However, the first two force fields are primarily employed for simulating biomolecules. The COMPASS force field is extensively utilized in covalent molecular systems, including a wide range of common organic molecules, small inorganic molecules, and polymers. COMPASS has been proven to be efficient in predicting the interactions of both organic and inorganic compounds [31,32]. The non-bonded interactions between atoms were described using long-range electrostatic interactions and short-range van der Waals (vdW) interactions. The electrostatic interactions were computed using Coulomb’s law, while the vdW interactions were calculated using the Lennard–Jones potential. In our simulations, periodic boundary conditions were applied in all directions for each simulation cell. The atom-based method was employed to calculate van der Waals interactions, while the Ewald method was utilized to handle long-range electrostatic interactions [33]. The cutoff radius for non-bonded interactions was set at 1.25 nm and the buffer width was set at 0.05 nm.

**Figure 4:**
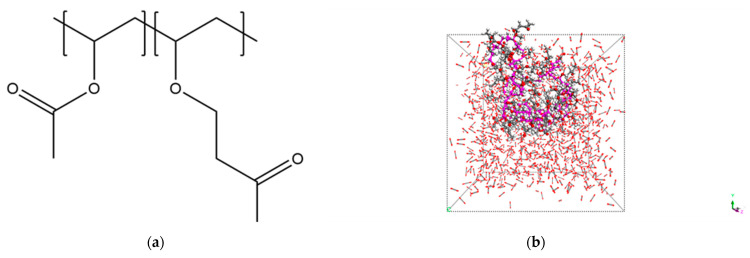
(**a**) The structural formula of polymer PVAO. (**b**) The snapshot of the simulation box.

The *Forcite* module was employed to perform structural optimization of the unit cell. The Smart Minimizer was utilized during the model structure optimization process. The lowest energy configuration was selected and annealed for 5 cycles within the temperature range of 300–500 K. Following annealing, NVT (constant number of particles, volume, and temperature) simulations for 300 ps and NPT (constant number of particles, pressure, and temperature) simulations for 300 ps were conducted, with a time step of 1 fs [31,34,35]. For both the NVT and NPT ensembles, we employed the Nose–Hoover method to implement the barostat for temperature and pressure control. The Q ratio was set to 0.01. The research conducted by D. J. Evans and B. L. Holian demonstrated that different thermostats have negligible effects on parameters such as shear viscosity and internal energy. The Nose–Hoover thermostat is commonly employed in both NVT and NPT ensembles to regulate the system temperature. The Nose–Hoover method, which strictly adheres to the canonical ensemble, is often utilized as a technique for equilibrium sampling [36]. Trajectories were saved at 5 ps intervals, and the configurations of the final 50 ps were used for data analysis. The parameters such as interaction energy and cohesive energy density were computed by averaging the values obtained from the last 10 frames. Subsequently, the thermodynamic parameters for the various systems were obtained [37,38]. The MD simulations were adopted to study PVAc and PVAO thickened scCO_2_ systems, of which the newly designed structure PVAO was shown in Figure 4a, and the design principle was explained below. A snapshot of the MD simulation for the PVAO/CO_2_ system is depicted in Figure 4b.

## 4. Design

Kazarian et al. proposed that the dissolution of polymers in scCO_2_ is primarily governed by the interactions between polymers and CO_2_, including Lewis acid–base (LA–LB) interactions and weaker hydrogen bonding [39]. Beckman et al. confirmed that the O atoms of carbonyl groups can increase the solubility of polymers in CO_2_ through LA–LB interactions, suggesting that the favorable dissolution behavior of PVAc may be attributed to the interactions between its carbonyl groups and CO_2_ [18]. Raveendran et al. demonstrated that there are also interactions between the H atoms adjacent to the C atoms in polymer molecules and the O atoms in CO_2_, which can be classified as hydrogen bonding [40]. Although such interactions are relatively weak, they can still enhance the interaction capability between polymer molecules and CO_2_. The interactions between polymers and CO_2_ primarily originate from functional groups, and identifying favorable functional groups is beneficial for designing new structures. According to the simulations conducted by Kilic et al., the interaction energies between the O atoms of ether groups and CO_2_ are of the same order of magnitude as those between carbonyl groups and CO_2_ [41]. Thus, ether groups are likely to play an active role in polymer–CO_2_ interactions, providing insight for the design of new structures in this study. Here, we proposed a newly designed configuration which was found to exhibit superior performance in viscosity enhancement compared to PVAc under comparable weight percentages, while maintaining better solubility characteristics than PVAc. Figure 4a illustrates the newly designed configuration of poly [(vinyl acetate)-(4-vinyl ethoxy butan-2-one)], hereafter referred to as PVAO.

The research conducted by Hu et al. demonstrated that the simulation results of polymers are influenced by the number of repeating units. It is observed that when the number of repeating units exceeds 30–40, the thermodynamic parameters become insensitive to the molecular weight [20,21]. Then, we selected a PVAc chain with a degree of polymerization of n = 75 (Mn = 6452 g/mol) and constructed a similarly sized PVAO chain with a degree of polymerization of n = 33 (Mn = 7002 g/mol). Table 3 presents five systems, including pure scCO_2_ with 1000 CO_2_ molecules, a system containing 1 PVAc chain with 1000 CO_2_ molecules, a system containing 1 PVAO chain with 1000 CO_2_ molecules, and systems containing 3 PVAO chains and 3 PVAc chains. Considering the temperature and pressure of well sites, temperatures ranging from 120 to 200 °C and pressures ranging from 60 to 120 MPa were selected.

Furthermore, to validate the rationality of the parameters used in the MD simulations, an scCO_2_ fluid model with 2.9 wt% PVAc content was constructed, and the relative viscosity was calculated to be 1.8 times that of pure scCO_2_ fluid at 23 °C and 20 MPa, which closely matches the experimental data that shows a relative viscosity of 1.7 times [42]. All of the parameters were chosen to be consistent with the aforementioned system.

## 5. Conclusions

ScCO_2_ has wide application in oil recovery, such as use as an oil-displacing agent in EOR. And improving its viscosity and solubility is one of the important research topics. Based on the structure of PVAc, a novel environmentally friendly polymer thickener was designed considering the interaction mechanisms of the functional groups in CO_2_, aiming to find polymers with enhanced affinity for CO_2_ through PVAc modifications.

Here, we proposed a newly designed polymer thickener named PVAO. MD simulations were conducted on the thickened scCO_2_ system to investigate the viscosity enhancement effects and solution characteristics systematically. Under identical temperature and pressure conditions, and similar weight percentages, PVAO turned out to exhibit superior viscosity enhancement compared to PVAc. The viscosity of scCO_2_ with a PVAO chain was approximately 1.5 times that of scCO_2_ with PVAc. Further MD simulations were performed on PVAO to obtain its radial distribution function, identifying the functional groups that contributed to the crucial interactions. It was found that stronger LA–LB interactions were observed between the carbonyl oxygen atom at the distal end of the PVAO side chain and the carbon atom in CO_2_, instead of the oxygen atom at the proximal end. The interaction energy, cohesive energy density, and solubility parameters of PVAO were obtained to analyze its dissolution capacity in CO_2_. It was found that, under the simulated temperature and pressure conditions, PVAO exhibited better dissolution capacity than PVAc. Thus, PVAO is a novel CO_2_-philicity polymer with higher viscosity-enhancement efficiency and better dissolution capacity than PVAc.

Comparatively, the viscosity enhancement and solubility of PVAO and PVAc were lower than fluorinated and siloxane-based polymers, and there is still a long way to go to improve the viscosity enhancement and solubility performance of polymers. Also, the synthesis pathway of PVAO still remains unclear. PVAO also faces potential challenges in oil production site application. Yet what counts is that they have merits over others considering environmental friendliness and economic efficiency aspects. Our findings offer insights for the design of other high-performance polymers and provide theoretical instruction for oil site applications.

## Figures and Tables

**Table 1:** Interaction Energy between CO_2_ and Polymer Single Chain at 120 °C and 60 MPa(Unit: kJ/mol).

Composition	$E_{CO_{2}-chain}$	$E_{CO_{2}}$	$E_{chain}$	$E_{inter}$
PVAc/CO_2_	−3140.5	−1162.8	−1503.0	−474.6
PVAO/CO_2_	−2273.9	−1772.0	−8.9	−493.1

**Table 2:** Cohesive energy density and solubility parameters under 120 °C and 60 MPa conditions.

Composition	$e_{coh}$ (J/m^3^)	δ ((J/m^3^)^1/2^)
CO_2_	2.025 × 10^8^	14.18
PVAc	2.561 × 10^8^	15.98
PVAO	2.326 × 10^8^	15.21

**Table 3:** Different polymer and CO_2_ systems in MD simulations.

System	Composition	No. of Chains	Mn of Chain	No. of VAc Units	No.of VO Units	No. of CO_2_	No. of Atoms	Size (nm)
1	CO_2_					1000	3000	4 × 4 × 4
2	PVAc/CO_2_	1	6452	75	0	1000	3902	4.2 × 4.2 × 4.2
3	PVAO/CO_2_	1	7002	35	35	1000	4052	4.2 × 4.2 × 4.2
4	PVAc	3	6452	75	0	0	2706	3 × 3 × 3
5	PVAO	3	7002	35	35	0	3156	3 × 3 × 3

## Data Availability

Data is contained within the article.
